# Association between preoperative extracellular water–to–total body water ratio and time to walking independence after total hip arthroplasty: a retrospective cohort study

**DOI:** 10.1007/s00264-026-06815-1

**Published:** 2026-04-29

**Authors:** Atsushi Shinonaga, Hiromi Matsumoto, Mana Uekawa, Saki Kakeya, Shuro Furuichi, Shigeru Mitani, Shigeharu Tanaka, Naoki Deguchi, Ryo Tanaka

**Affiliations:** 1 Graduate School of Humanities and Social Sciences, Hiroshima University, Higashi-Hiroshima, Japan; 2 Rehabilitation Center, Kawasaki Geriatric Medical Center, Okayama, Japan; 3 Department of Physical Therapy, Faculty of Rehabilitation, Kawasaki University of Medical Welfare, Kurashiki, Japan; 4 Rehabilitation Center, Kawasaki Medical School Hospital, Kurashiki, Japan; 5 Department of Spine and Joint Surgery, Kawasaki Medical School, Kurashiki, Japan; 6 Physical Therapy Major, Department of Rehabilitation, Faculty of Health Sciences, Tokyo Kasei University, Sayama, Japan; 7 Department of Integrated Health Sciences, Prevention and Rehabilitation Sciences, Graduate School of Medicine, Nagoya University, Nagoya, Japan

**Keywords:** Total hip arthroplasty, Extracellular water–to–total body water ratio, Walking independence, Muscle quality

## Abstract

**Purpose:**

To investigate the association between preoperative extracellular water–to–total body water ratio (ECW/TBW) and time to walking independence among female patients undergoing total hip arthroplasty (THA).

**Methods:**

This retrospective cohort study included female patients who underwent primary THA between January and December 2022. Preoperative ECW/TBW was measured using bioelectrical impedance analysis and dichotomized at 0.400. The primary outcome was time to walking independence within 14 postoperative days. Cox proportional hazards models assessed the association between ECW/TBW and walking independence, adjusting for age, comorbidities, nutritional status, skeletal muscle mass index, non-operated knee extensor strength, walking pain, and maximum walking speed. Model performance was evaluated using likelihood ratio tests, Harrell’s C-index, and time-dependent area under the curve (AUC).

**Results:**

Among 142 patients, 118 (83.1%) achieved walking independence within 14 days. Patients with ECW/TBW ≥ 0.400 achieved walking independence later than those with ECW/TBW < 0.400 (log-rank p < 0.001). In multivariable analysis, ECW/TBW ≥ 0.400 was associated with delayed walking independence (hazard ratio 0.29, 95% CI 0.16–0.52). Including ECW/TBW improved model fit and increased the time-dependent AUC at postoperative day 14.

**Conclusion:**

Higher preoperative ECW/TBW is associated with delayed walking independence after THA and may complement preoperative assessment when predicting early postoperative functional recovery.

**Supplementary Information:**

The online version contains supplementary material available at https://doi.org/10.1007/s00264-026-06815-1.

## Introduction

Recovery of mobility after total hip arthroplasty (THA) is a key functional outcome in perioperative care. As the number of THA procedures increases worldwide [1], prolonged hospitalization remains a concern due to its impact on healthcare costs [2]. Postoperative mobility is closely associated with length of stay, and delayed recovery may contribute to prolonged hospitalization [3]. Walking independence is a central component of mobility, with substantial interindividual variability; approximately 30% of patients fail to achieve it within two weeks after THA [4]. Persistent difficulty in walking is also associated with a reduced likelihood of discharge home [5]. Identifying patients at risk for delayed walking independence is therefore important for optimizing perioperative management and reducing prolonged hospitalization.

Accurate assessment of preoperative status is essential for predicting the attainment of walking independence after THA and for optimizing perioperative management. Previous studies have demonstrated that lower preoperative knee extensor muscle strength is associated with a reduced likelihood of achieving postoperative walking independence [4, 6]. Similarly, preoperative maximum walking speed has been reported as a simple and practical predictor of postoperative walking independence [7]. Preoperative malnutrition, as assessed by the Controlling Nutritional Status (CONUT) score, has also been associated with delayed recovery of walking independence after THA [8]. Collectively, these findings indicate that preoperative physical function and nutritional status provide important insights into postoperative mobility recovery following THA.

In recent years, body fluid distribution has been proposed as an indicator of physical function and nutritional status, although its role in preoperative assessment for THA remains unclear. It comprises intracellular water (ICW) and extracellular water (ECW) and can be measured using bioelectrical impedance analysis (BIA) [9]. The extracellular water–to–total body water ratio (ECW/TBW) reflects body water balance and edema status [9] and has been associated with impaired physical function in older adults, reflecting muscle quality rather than muscle mass [10, 11]. It has also been negatively associated with recovery of activities of daily living in hospitalized older individuals [12]. Given these findings, ECW/TBW may be relevant to walking independence after THA; however, its characteristics in this population and its association with postoperative walking independence remain unclear. Clarifying this relationship may improve preoperative risk stratification and support individualized rehabilitation planning.

This study aimed to investigate the association between preoperative ECW/TBW and time to walking independence among female patients undergoing THA. Because women comprise the majority of patients undergoing THA [13], this study focused on women. We also examined whether inclusion of ECW/TBW improved model fit and discrimination beyond conventional clinical factors. We hypothesized that higher preoperative ECW/TBW would be associated with delayed postoperative walking independence.

## Materials and methods

### Study design and setting

In this retrospective cohort study, we investigated the association between preoperative ECW/TBW and time to attainment of walking independence after THA. This study was conducted at a single institution in Japan. The study period was from January to December 2022.

## Participants

Inclusion criteria were female sex, age > 40 years, hip osteoarthritis, and admission for primary THA. Exclusion criteria were pacemaker implantation, other internal metal implants, postoperative THA complications (e.g., dislocation, fracture, nerve injury), and non-Japanese nationality. Patients with pacemakers or other metal implants were excluded because BIA is contraindicated in implanted cardiac devices and may be affected by metal implants [9]. As the study was conducted in Japan, the analysis was restricted to Japanese participants.

## Postoperative protocol

During the study period, six experienced surgeons (median years of experience, 20 (10–35) years) performed THA. Postoperative rehabilitation was initiated with the goal of achieving stable ambulation with a cane within approximately two weeks, provided no major complications occurred. Physical therapy, including progressive gait training and lower extremity strengthening, was conducted five to six times per week for 40–60 min per session.

## Variables

### Outcome

The primary outcome was time to walking independence after THA. Walking independence was defined as the ability to walk 50 m independently, with or without a walking aid, based on the Functional Independence Measure [14]. It was assessed daily during hospitalization by consensus between the attending physical therapist and nurse. Time to walking independence was analyzed as a time-to-event outcome over a 14-day postoperative period, reflecting the typical hospital stay for THA in Japan [15].

### Exposures

Preoperative ECW/TBW was measured using bioelectrical impedance analysis (InBody 770; InBody Japan Co., Ltd., Tokyo, Japan) on the day before THA. This device uses direct segmental multifrequency bioelectrical impedance analysis (DSM-BIA), whose validity and reliability have been previously reported [16]. Measurements were performed in the standing position at least two hours after a meal. ECW/TBW was calculated as the ratio of extracellular to total body water. ECW/TBW was dichotomized at 0.400, corresponding to the upper limit of the normal range (0.360–0.400) [17, 18] and commonly used to define excess fluid retention [19]. This cutoff has also been used in previous studies [20, 21].

### Potential confounders

Demographic factors (age, height, weight, and body mass index), hip osteoarthritis (OA) severity, surgical approach, other musculoskeletal diseases, medical comorbidities, and the CONUT score were obtained from medical charts at hospitalization. OA severity was assessed preoperatively using the Kellgren–Lawrence grading system [22]. Surgical approach (anterolateral or posterolateral) was recorded. Other musculoskeletal diseases included contralateral hip OA, knee OA, and spinal disorders. Comorbidities were evaluated using the modified Charlson Comorbidity Index (CCI) [23]. The CONUT score, calculated from blood tests obtained approximately two weeks before THA, is based on serum albumin, total lymphocyte count, and total cholesterol, with higher scores indicating poorer nutritional status [24]. Patients were classified as having normal nutritional status (CONUT 0–1) or malnutrition (CONUT ≥ 2) [8].

In addition to the above variables, preoperative physical function was assessed by a physical therapist on the day before THA, including bilateral knee extensor strength, maximum walking speed, and pain during walking. Skeletal muscle mass index (SMI) was measured using the same bioelectrical impedance analysis device. Knee extensor strength was assessed as maximal isometric strength using a handheld dynamometer (μ-Tas F-1; Anima, Tokyo, Japan) with a fixation strap. Participants were seated with the hip and knee at 90°, and two measurements per limb were averaged. Force was converted to torque and normalized to body weight (Nm/kg) [25]. Maximum walking speed was assessed over an 11-m walkway, with timing over the central 5 m; two trials at the fastest safe speed were averaged [26]. Pain during walking was evaluated using a 100-mm visual analog scale [27].

## Bias

Consecutive patients who underwent THA during the inclusion period were enrolled to minimize selection bias. Due to the retrospective design, complete blinding was not feasible; however, ECW/TBW results were not disclosed to physical therapists and nurses assessing postoperative walking independence, reducing the risk of assessment bias.

## Statistical analysis

Baseline characteristics were summarized as means with standard deviations for continuous variables and as counts with percentages for categorical variables. Kaplan–Meier survival curves were constructed to describe time to walking independence, stratified by ECW/TBW group (< 0.400 vs. ≥ 0.400), and between-group differences were assessed using the log-rank test. Kaplan–Meier analysis included only patients with available ECW/TBW data.

Cox proportional hazards models were used to examine factors associated with time to walking independence. Missing data were handled using multiple imputation by chained equations with predictive mean matching [28]. All available variables were included in the imputation model, and 20 imputed datasets were generated. Regression analyses were performed separately for each dataset, and results were pooled using Rubin’s rules [29, 30]. Model 1 (base model) included age, CCI, CONUT score, SMI, non-affected side knee extensor muscle strength, pain during walking, and maximum walking speed. These variables were selected a priori based on prior studies reporting associations with postoperative walking independence [4, 6–8]. Non-affected side knee extensor muscle strength was included because previous evidence has demonstrated its superior predictive value for early postoperative walking independence compared with the affected side [6]. Model 2 (extended model) included all variables in Model 1, with the addition of ECW/TBW. To assess the robustness of the results independent of the predefined cutoff, ECW/TBW was entered as a continuous variable in a sensitivity analysis.

The incremental contribution of ECW/TBW to model fit was evaluated using the likelihood ratio test. Overall discrimination was assessed using Harrell’s concordance statistic (C-index), and differences between models were evaluated by nonparametric bootstrap resampling (1,000 iterations). Time-specific discrimination was assessed using time-dependent ROC analysis at postoperative days 7 and 14, with differences in AUC calculated at each time point. Day 7 was selected as an early recovery milestone, when approximately half of patients achieve walking independence after THA [4], and day 14 corresponded to the prespecified observation period reflecting hospital stay in Japan [15]. Uncertainty in ΔAUC was evaluated using bootstrap resampling with 95% confidence intervals. Analyses were performed using RStudio (Version 2025.09.0 + 387; Posit Software, PBC) and R (version 4.5.1), with dplyr for data processing, mice for multiple imputation, survival for survival analyses, and timeROC for time-dependent ROC analysis. A two-sided p < 0.05 was considered statistically significant.

## Sample size

Based on previous recommendations of five to ten events per variable for Cox regression [31], and with eight variables planned for inclusion, 40–80 events were required. Assuming a 70% event rate for walking independence within two weeks after THA [4], this corresponded to an estimated sample size of 57–114 participants. To ensure statistical stability and account for potential missing data and sensitivity analyses, we conservatively set the required sample size at ≥ 115 participants.

## Results

**Fig. 1:**
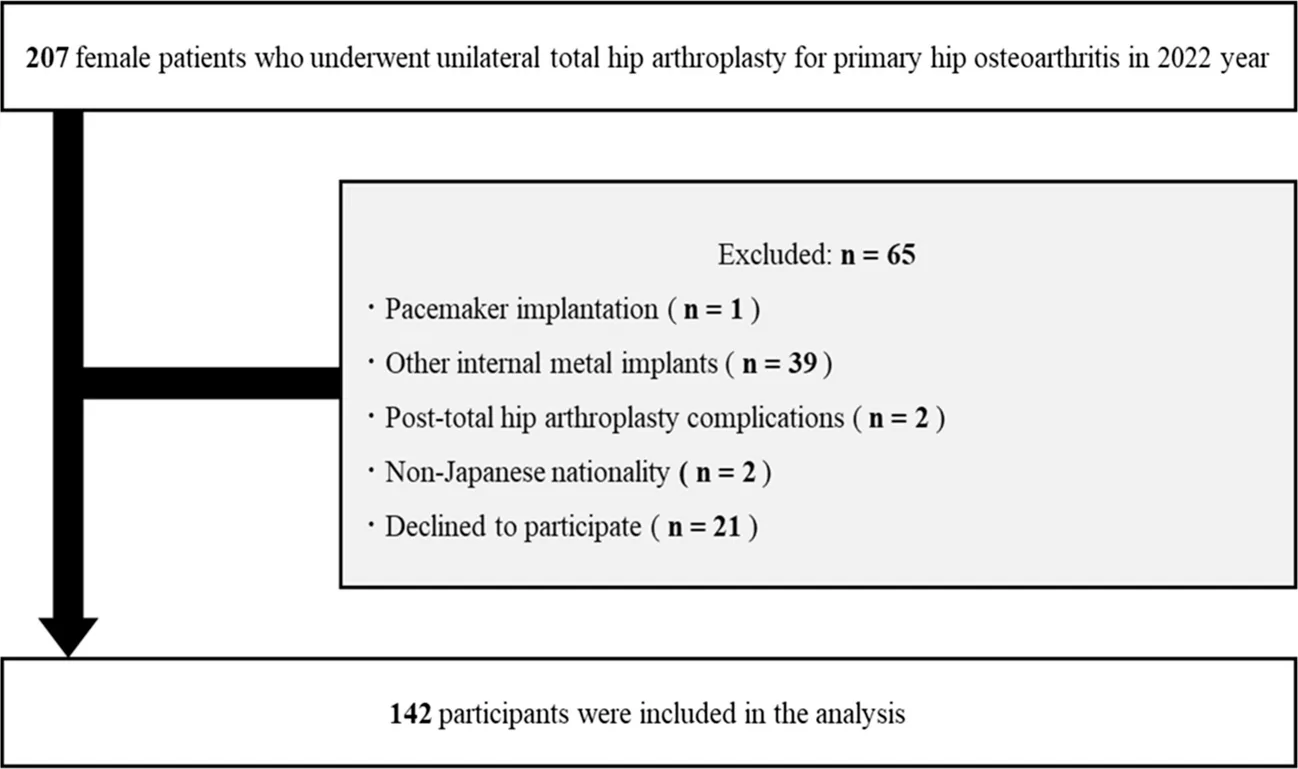
Flowchart of participants

During the study period, 207 female patients underwent THA, of whom 142 met the inclusion criteria and were included in the baseline analysis (Fig. 1). Baseline characteristics are shown in Table 1. The mean age was 67.1 ± 9.1 years, and 77.5% had Kellgren–Lawrence grade IV hip osteoarthritis. The mean ECW/TBW was 0.395 ± 0.007, with 32 patients (24.4%) having values ≥ 0.400. By day 14, 118 patients (83.1%) achieved walking independence.

**Table 1:** Baseline characteristics of the participants

	n	Values
Age (years)	142	67.1 ± 9.1
Height (cm)	142	153.5 ± 6.3
Weight (kg)	142	57.6 ± 10.3
Body mass index (kg/m^2^)	142	24.4 ± 4.0
Kellgren–Lawrence grade, n (%)	142	
Grade Ⅱ		3 (2.1)
Grade Ⅲ		29 (20.4)
Grade Ⅳ		110 (77.5)
Surgical approach, n (%)	142	
Posterolateral approach		35 (24.7)
Anterolateral approach		107 (75.3)
Musculoskeletal diseases, n (%)	142	
Contralateral hip osteoarthritis		23 (16.2)
Knee osteoarthritis		13 (9.2)
Spinal disease		19 (13.4)
Charlson comorbidity index	142	0.3 ± 0.7
Serum albumin (g/dL)	142	4.2 ± 0.3
Total lymphocytes (/mL)	142	1740.0 ± 666.2
Total cholesterol (mg/dL)	142	205.2 ± 33.9
CONUT score, n (%)	142	
Normal		104 (73.2)
Light malnutrition		38 (26.8)
Moderate malnutrition		0 (0.0)
Severe malnutrition		0 (0.0)
ECW/TBW	131	0.395 ± 0.007
ECW/TBW ≥ 0.400, n (%)	131	32 (24.4)
Skeletal muscle mass index (kg/m^2^)	131	6.0 ± 0.7
Knee extensor muscle strength (Nm/kg)		
Affected side	118	0.77 ± 0.29
Non-affected side	118	0.95 ± 0.33
Maximum walking speed (m/s)	128	1.09 ± 0.37
Pain during walking (mm)	128	49.4 ± 27.3
Walking independence by day 14, n (%)	142	118 (83.1)
Length of hospital stay (day)	142	18.6 ± 6.4

Values are presented as mean ± standard deviation or n (%). Means and percentages were calculated based on the number of patients with available data

n, number; cm, centimeter; kg, kilogram; m, meter; g/dL, grams per deciliter; m/mL, per milliliter; mg/dL, milligrams per deciliter; CONUT score, Controlling Nutritional Status score; ECW/TBW, extracellular water-to-total body water ratio; Nm, newton meter; m/s, meters per second; mm, millimeter

**Fig. 2:**
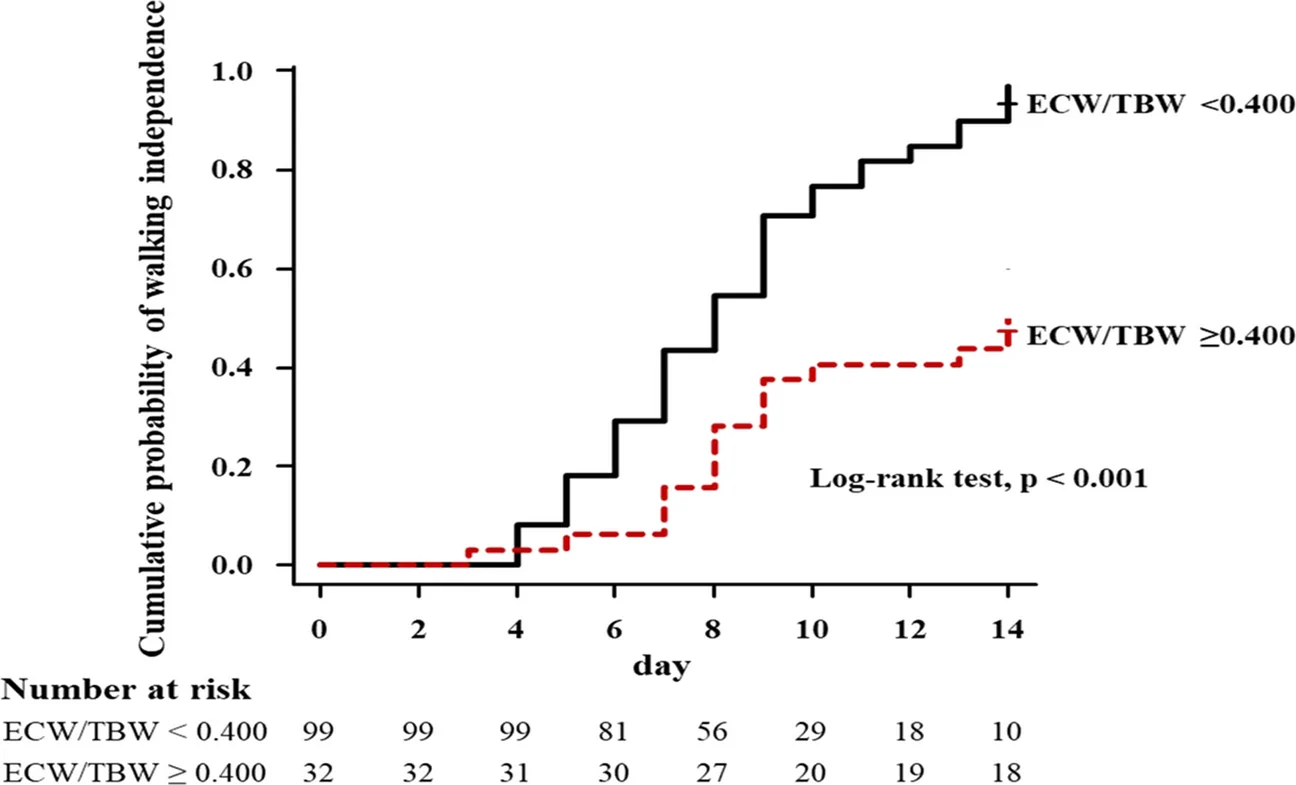
Kaplan–Meier analysis of time to walking independence according to ECW/TBW (< 0.400 vs. ≥ 0.400)

Kaplan–Meier survival curves for time to attainment of walking independence stratified by ECW/TBW (< 0.400 vs. ≥ 0.400) are presented in Fig. 2. ECW/TBW data were available for 131 patients, who were included in this analysis. Patients with ECW/TBW ≥ 0.400 achieved walking independence significantly later than those with ECW/TBW < 0.400 (log-rank test, p < 0.001).

The results of the multivariable Cox proportional hazards analyses are shown in Table 2. In the base model, maximum walking speed was associated with time to walking independence, with faster speed corresponding to a shorter time to independence (HR, 1.11; 95% CI, 1.05–1.18). After adding ECW/TBW, non-operated side knee extensor strength (HR, 1.08; 95% CI, 1.01–1.15) and maximum walking speed (HR, 1.08; 95% CI, 1.02–1.15) were both associated with time to walking independence. In this model, ECW/TBW ≥ 0.400 was associated with a longer time to independence (HR, 0.29; 95% CI, 0.16–0.52). Sensitivity analysis treating ECW/TBW as a continuous variable yielded consistent results (Supplementary Table 1).

**Table 2:** Cox proportional hazards regression analysis for walking independence

	Model 1			Model 2		
	HR	95%CI	*p*-value	HR	95%CI	*p*-value
Age	0.99	0.97–1.01	0.320	0.99	0.97–1.01	0.468
Charlson comorbidity index	1.00	0.73–1.37	0.989	1.09	0.79–1.50	0.619
CONUT score ≥ 2	0.89	0.57–1.38	0.603	0.91	0.58–1.43	0.687
Skeletal muscle mass index	0.99	0.96–1.02	0.362	0.98	0.95–1.01	0.236
Non-affected side knee extensor strength	1.06	0.99–1.13	0.091	1.08	1.01–1.15	0.021
Pain during walking	1.00	0.99–1.01	0.639	1.00	0.99–1.01	0.630
Maximum walking speed	1.11	1.05–1.18	< 0.001	1.08	1.02–1.15	0.014
ECW/TBW ≥ 0.400				0.29	0.16–0.52	< 0.001

Cox proportional hazards regression was conducted using pooled estimates from 20 imputed datasets based on Rubin’s rules

Continuous variables with small absolute ranges were rescaled for ease of interpretation. Hazard ratios are expressed per 0.1 kg/m^2^ increment in Skeletal muscle mass index, per 0.1 Nm/kg increment in non-affected knee extensor strength and per 0.1 m/s increment in maximum walking speed

No statistically significant violation of the proportional hazards assumption was observed

HR, hazard ration; CI, confidence interval; CONUT score, Controlling Nutritional Status score; ECW/TBW, extracellular water-to-total body water ratio

To examine the incremental predictive value of ECW/TBW, the base and extended models were compared. Inclusion of ECW/TBW improved model fit (likelihood ratio test: χ^2^ = 21.05, df = 1, p < 0.001). Overall discrimination increased, with the C-index rising from 0.68 to 0.71; however, the difference (ΔC = 0.032) was not statistically significant (95% CI, − 0.003–0.078). Time-dependent AUC increased in the extended model at day seven (0.76 to 0.80; mean ΔAUC, 0.038; 95% CI, − 0.019–0.110) and day 14 (0.81 to 0.88; mean ΔAUC, 0.063; 95% CI, 0.003–0.142), with significance reached at day 14.

## Discussion

In this study, higher preoperative ECW/TBW was associated with delayed postoperative walking independence in female patients undergoing THA. This association remained significant after adjustment for age, CCI, CONUT score, SMI, knee extensor strength on the non-affected side, pain during walking, and maximum walking speed. Furthermore, adding ECW/TBW to the multivariable model improved model fit and enhanced discrimination at specific postoperative time points. These findings suggest that preoperative ECW/TBW may provide complementary prognostic information beyond conventional clinical measures for predicting recovery of walking independence after THA.

The novelty of this study lies in examining the association between preoperative ECW/TBW and early walking independence after THA. Previous studies have linked ECW/TBW to impaired physical function, sarcopenia in older adults, and delayed recovery of activities of daily living in hospitalized patients [10–12]. However, to our knowledge, this association has not been examined in patients undergoing THA, particularly in relation to early postoperative walking independence. Thus, this study positions ECW/TBW within the context of early postoperative walking independence after THA.

The findings suggest that delayed walking independence in patients with higher preoperative ECW/TBW may be related to altered muscle quality or fluid imbalance–related functional limitations. Elevated ECW/TBW has been reported as a marker of muscle quality independent of muscle mass and is associated with poorer physical function, including reduced strength and slower walking speed [32]. It also reflects body fluid balance and oedema status [33]. In related populations, such as patients with hip fracture and hospitalized older adults, greater oedema has been associated with poorer improvements in muscle strength, reduced mobility, and prolonged hospitalization [34, 35]. These findings suggest that preoperative ECW/TBW may help identify patients at risk of delayed walking independence after THA.

The present findings suggest that preoperative ECW/TBW may complement conventional measures in assessing early postoperative walking independence. Inclusion of ECW/TBW improved model fit, indicating that body fluid distribution contributes beyond conventional predictors. Although overall discrimination across the follow-up period showed limited improvement, time-dependent ROC analysis demonstrated a significant increase at postoperative day 14, suggesting a greater contribution of ECW/TBW around discharge. This improvement may partly reflect a clearer independent association between non-operated side knee extensor strength and time to walking independence after including ECW/TBW. Accounting for body fluid distribution may thus reduce confounding effects on physical function measures and improve their clinical interpretation. Previous studies have reported that elevated ECW/TBW can obscure associations between bioelectrical impedance–derived physical function measures, supporting this interpretation [20]. Overall, ECW/TBW appears to serve as complementary information rather than a stand-alone predictor when estimating walking independence approximately two weeks after THA.

Several limitations should be considered. First, the retrospective single-center design precludes causal inference; thus, the associations observed between ECW/TBW, physical function, and time to walking independence should be interpreted as correlational. Second, ECW/TBW was measured only preoperatively, and postoperative changes in body fluid distribution were not assessed. Third, this study was not intended to develop or validate a predictive model; no internal or external validation was performed, and analyses were exploratory. Finally, as only female patients were included, generalizability to males may be limited. Despite these limitations, the findings suggest that preoperative ECW/TBW may provide complementary information for assessing early postoperative walking independence after THA.

In conclusion, higher preoperative ECW/TBW was associated with delayed walking independence after THA. Its inclusion improved model fit beyond conventional clinical factors, suggesting that ECW/TBW may provide complementary information for assessing early postoperative recovery.

## Supplementary Information

Below is the link to the electronic supplementary material.Supplementary file1 (DOCX 33 KB)

## Data Availability

The surveys and materials are available upon reasonable request to the corresponding author.
