# Supplementary material for: Association between preoperative extracellular water–to–total body water ratio and time to walking independence after total hip arthroplasty: a retrospective cohort study
Source: Int Orthop. 2026 Apr 29;50(8):1881–8. doi: 10.1007/s00264-026-06815-1 (PMC13525042; doi:10.1007/s00264-026-06815-1)
Supplement: Supplementary file 1 [file 264_2026_6815_MOESM1_ESM.docx]

| **Supplementary Table 1. Cox proportional hazards regression analysis for walking independence in sensitivity analysis** | | | | | | |
| --- | --- | --- | --- | --- | --- | --- |
|  | Model 1 | | | Model 2 | | |
|  | HR | 95%CI | *p*-value | HR | 95%CI | *p*-value |
| Age | 0.99 | 0.97–1.01 | 0.320 | 0.99 | 0.97–1.01 | 0.497 |
| Charlson comorbidity index | 1.00 | 0.73–1.37 | 0.989 | 1.19 | 0.85–1.67 | 0.301 |
| CONUT score ≥2 | 0.89 | 0.57–1.38 | 0.603 | 0.84 | 0.54–1.31 | 0.438 |
| Skeletal muscle mass index | 0.98 | 0.96–1.02 | 0.362 | 0.98 | 0.95–1.00 | 0.108 |
| Non-affected side knee extensor strength | 1.06 | 0.99–1.13 | 0.091 | 1.08 | 1.01–1.15 | 0.021 |
| Pain during walking | 1.00 | 0.99–1.01 | 0.639 | 1.00 | 0.99–1.01 | 0.611 |
| Maximum walking speed | 1.11 | 1.05–1.18 | <0.001 | 1.07 | 1.01–1.14 | 0.028 |
| ECW/TBW |  |  |  | 0.01 | 0.00–0.09 | <0.001 |
| Cox proportional hazards regression was conducted using pooled estimates from 20 imputed datasets based on Rubin’s rules.  In this sensitivity analysis, ECW/TBW was entered as a continuous variable.  Continuous variables with small absolute ranges were rescaled for ease of interpretation. Hazard ratios are expressed per 0.1 kg/m^2^ increment in Skeletal muscle mass index, per 0.1 Nm/kg increment in non-affected knee extensor strength, per 0.1 m/s increment in maximum walking speed and per 0.1 increment in ECW/TBW.  HR, hazard ration; CI, confidence interval; CONUT score, Controlling Nutritional Status score; ECW/TBW, extracellular water-to-total body water ratio | | | | | | |
